# Clinical relevance of heparin-PF4 complex antibody in DVT after total joint replacement

**DOI:** 10.1186/1471-2474-10-42

**Published:** 2009-04-28

**Authors:** Takafumi Torigoshi, Satoru Motokawa, Yumi Maeda, Kazushige Maeda, Takeshi Hiura, Gou Takayama, Kenji Taguchi, Hiroyuki Shindo, Kiyoshi Migita

**Affiliations:** 1 Department of Orthopedics Surgery and Department of Rheumatology, NHO Nagasaki Medical Center, Nagasaki, Japan; 2 Clinical Research Center, NHO Nagasaki Medical Center, Kubara 2-1001-1, Omura, 856-8652, Japan; 3 Department of Orthopedics Surgery, Nagasaki University School of Medicine Sakamoto 1-7-1, Nagasaki, 852-8501, Japan

## Abstract

**Background:**

Antibodies to the heparin-platelet factor-4 (HPF-4) complex (HIT antibodies) have been observed in patients with heparin-induced thrombocytopenia (HIT). These antibodies are thought to be involved in thrombosis through activation of platelet/endothelial cells. This prospective study was conducted to determine the incidence of post-operative HIT antibodies to assess the associated risk of deep vein thrombosis (DVT) in patients undergoing total knee arthroplasty (TKA) or total hip arthroplasty (THA).

**Methods:**

We studied 104 patients who underwent unilateral primary TKA (n = 44) and primary THA (n = 60) with short-duration prophylaxis (1–2 days of a fixed dose of unfractionated heparin). HIT antibodies were assayed using a sandwich-type ELISA before the operation and after heparin treatment (post-operative day 7).

**Results:**

In the clinical outcome, the incidence of symptomatic DVT was 15.4% (16/104, TKA; 10, THA 6) and pulmonary embolism (PE) was not observed. The total seroconversion rate of HIT antibodies at post-operative day 7 was 34.6% (36/104). Among 36 seroconverted patients, 11 (30.6%) developed symptomatic DVT and 5 out of 68 of the non-seroconverted patients (7.4%) developed symptomatic DVT. The incidence for DVT was significantly higher in the seroconverted patients compared with that of the non-seroconverted patients (odds ratio 5.5, 95%CI: 1.7–17.6 *p *= 0.0028). Furthermore, in the patients with symptomatic DVT, the titer of HIT antibodies at post-operative day 7 was significantly higher compared with those without symptomatic DVT.

**Conclusion:**

Our data therefore suggest that seroconversion for HIT antibodies generated by heparin is associated with a risk of DVT in patients undergoing total joint replacement.

## Background

Venous thromboembolism (VTE) is a common complication of surgical procedures. The risk for VTE in surgical patients is determined by the combination of individual predisposing factors and the specific type of surgery [1]. Patients undergoing major orthopedic surgery, which includes hip and knee arthroplasty, are at particularly high risk for VTE [2]. Postoperative DVT of the lower limbs is often asymptomatic, and a fatal pulmonary embolism (PE) may be the first clinical manifestation of postoperative VTE [3]. Therefore, routine screening for DVT of the lower limbs and early treatment are required. In patients undergoing total joint replacement in the absence of any prophylaxis, the incidence of venography-detected DVT ranges from 42% to 57% in THA and 41% to 85% in TKA [4], while that of clinically overt PE ranges between 0.1% and 1.0% [5].

A number of anticoagulant-based regimens have been evaluated for the prophylaxis of VTE in patients undergoing joint replacement surgery. A randomized trial has shown that prophylaxis with unfractionated heparin (UFH) is more effective than no prophylaxis in patients undergoing hip replacement [6]. Prophylaxis with UFH reduces the occurrence of DVT, but has been associated with an increased incidence of heparin-induced thrombocytopenia (HIT) [7]. HIT is an adverse drug reaction caused by the generation of an antibody against platelet factor-4 (PF4) bound to heparin, which activates platelets [8]. The frequency of HIT is about 3~5% in orthopedic surgery patients treated with UFH [9]. However, the exact role of HIT antibodies in DVT associated with orthopedic surgery has not been fully elucidated. Recently, the presence of HIT antibodies has been reported to predict adverse events in post-operative patients receiving heparin [10]. In this study, we evaluated the levels of HIT antibodies in patients undergoing THA or TKA under UFH prophylaxis in relation to the DVT risk.

## Methods

### Patients

All patients who underwent primary TKA and primary THA between September 1, 2006 and September 31, 2007 at our institution were enrolled in this study to determine the incidence of PE and symptomatic DVT. In this study, we enrolled 104 subjects (17 males and 87 females, 44 knee joints, 60 hip joints, age range 33–89 years, mean age 68.0 years). The underlying disease was osteoarthritis (OA) in 86 joints and rheumatoid arthritis (RA) in 18 joints (Table 1). All patients received 1000 units of UFH via a single bolus intravenous injection during the operation and 5000 units of UFH via drip intravenous infusion (24 hr) at post-operative day 2 after the operation. The operation was performed under general anesthesia in all cases. An extremity tourniquet was routinely used during surgery to control blood loss in TKA. A foot pump (A-V Impulse System, Novamedix Corp, Hampshire, UK) was started on day 1 in all subjects. None of the patients had any history of previous heparin exposure within the past 90 days. The study protocol was approved by the Ethics Committees of the Nagasaki Medical Center and written informed consent was obtained from each patient.

**Table 1:** Patients characteristics

	**Type of surgery**		
		
**Characteristics**	**THA(n = 60)**	**TKA(n = 44)**	***p-value***
Age (years), mean (SD)	63.1(12.1)	74.6(7.9)	< 0.01
Gender (male/female)	8/52	9/35	0.33
BMI (Kg/m^2^), mean (SD)	24.3(4.0)	25.9(4.7)	0.13
Metabolic diseases, n(%)	20(33.3%)	23(52.3%)	0.05
Hypertension			
Diabetes			
Hyperlipidemia			
History of thrombosis	0	1(2.3%)	-
Primary diseases	8/52	10/34	0.26
(RA/OA)			

DVT, deep vein thrombosis; SD, standard deviation; BMI, body mass index;

TKA, total knee arthroplasty; THA, total hip arthroplasty;

RA, rheumatoid arthritis; OA, osteoarthritis.

### Blood sampling

Serum samples were collected before operation and at postoperative day 7, and stored at -70°C. A sandwich ELISA kit (GTI Diagnostics, Waukesha, WI) was used to measure the anti-heparin-PE4 antibody (HIT Ab) titer of the serum according to the manufacturer's instructions, with the exception of the cut-off values. The microtiter wells were precoated with polyvinylsulfonate-PF4 complexes. Antibodies bound to polyvinylsulfonate-PF4 were identified by affinity-purified anti-human IgG, IgA, or IgM peroxidase conjugate. ELISA reactivities (optical density, OD) are expressed relative to a standard control. Positive and negative controls were included in every plate. The samples with readings greater than or equal to 0.55 absorbance units (mean+2SD of 40 sera from Japanese healthy subjects) were considered positive. Inhibition of a positive reaction by 50% or more in the presence of excess heparin was confirmed in all HIT antibody seroconverted patients. While the results of the ELISA test merely confirm the presence of HIT antibodies, HIT was defined as a >50% reduction in the platelet count or an absolute platelet count of < 10 × 10^4^/ml during and after heparin treatment, with no other cause of thrombocytopenia, and a positive result in the HIT antibody test [11]. For measurement of D-dimer, venous blood was collected in 5-mL Vacutainer^® ^tubes prefilled with 0.5 mL of 3.2% (0.105 mol/L) trisodium citrate-dihydrate. Specimens were centrifuged at 1700 *g *for 15 minutes at room temperature. D-dimer assays were performed according to the manufacturer's instructions on a CA-1500 automated coagulometer (Sysmex, Kobe, Japan) using a commercial kit (Afresa Pharma Co, Osaka, Japan).

### Assessment of venous thromboembolism

The patients were clinically examined daily for signs and symptoms of deep vein thrombosis, including pain, swelling, redness of the legs and positivity for Homan's sign, or pulmonary embolism until post-operative day 25-day 30. If these were present, bilateral venography or contrast-enhanced computed tomography were performed. Venography or computed tomography was evaluated by an independent panel of radiologists. The thrombi were classified as proximal if the popliteal or femoral veins were involved and distal if only the calf veins were implicated.

### Assessment of bleeding

During this study, the patients were examined daily for any evidence of wound hematoma, as well as other manifestations of bleeding. Bleeding was considered major if there was a wound hematoma necessitating surgical revision, gastrointestinal bleeding, or any bleeding requiring blood transfusion and interruption of prophylaxis.

### Statistical analysis

A comparison of the variables in the two groups, with or without DVT, was performed by Fisher's exact test or Mann-Whitney *U*-test. We used Fisher's exact test to calculate the 95% CI for the absolute difference and risk ratio between patients with or without HIT antibodies.

## Results

### Postoperative outcome

No major bleeding complications were observed in any of the 104 patients who underwent surgery, and no mortality occurred within the 3 post-operative months. No complications were associated with the use of intraoperative heparin or the pneumatic compression device, and none of the patients developed symptomatic PE. Patients with clinical symptoms or signs that would lead us to suspect DVT were investigated using either contrast-enhanced CT or venography. These examinations confirmed the clinical diagnosis of symptomatic DVT in 16 patients (TKA; 10/44, THA; 6/60). These DVT occurred during post-operative day5~day14. No proximal thrombi were noted in any of the patients. The patient's preoperative profiles are listed in Table 2. The profiles were similar in gender, laboratory data, and risk factors in patients who were positive or negative for post-operative symptomatic DVT, although not for the patient age.

**Table 2:** Patient characteristics and symptomatic DVT

**Characteristics**	**DVT(-)** **n = 88**	**DVT(+)** **n = 16**	***p-value***
Age (years), mean (SD)	66.9 (12.5)	73.8 (5.4)	0.04
Gender (male/female)	16/72	1/15	0.24
BMI (Kg/m^2^), mean (SD)	24.9 (4.4)	25.7 (4.1)	0.42
Metabolic diseases, n(%)	36 (40.9)	7 (43.8)	0.83
Hypertension			
Diabetes			
Hyperlipidemia			
History of thrombosis	0	1 (6.3)	-
TKA/THA	46/42	10/6	0.08
Primary diseases	16/72	2/14	0.54
(RA/OA)			

DVT, deep vein thrombosis; SD, standard deviation; BMI, body mass index;

TKA, total knee arthroplasty; THA, total hip arthroplasty;

RA, rheumatoid arthritis; OA, osteoarthritis.

### HIT antibody status

**Figure 1:**
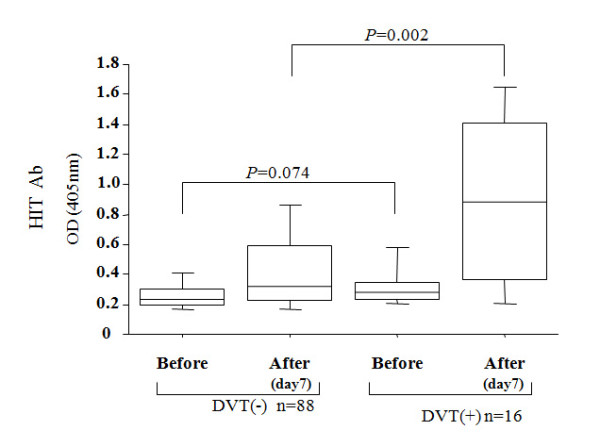
**Median optical density (OD) of HIT antibodies before and after operation (post-operative day 7) in patients receiving joint arthroplasty**. Box plots indicate the mean, interquartile rage (25%–75%) and outliers (min, max).

The HIT antibodies were present in 34.6% (36/104) of the total patients. As shown in Table 3, the risk for postoperative symptomatic DVT was significantly higher in patients with HIT antibodies compared with those without HIT antibodies (OR; 5.5; 95% CI 1.7–17.6). The overall HIT antibody optical densities were compared between the patients with or without symptomatic DVT. No significant differences in the HIT antibody optical densities before operation were noted between the patients with or without symptomatic DVT. However, the patients with symptomatic DVT demonstrated significantly higher HIT antibody optical densities at post-operative day 7, compared with those without symptomatic DVT (Figure 1).

**Table 3:** Comparison of symptomatic DVT incidence rate between seroconverted and non-converted patients

	**Seroconverted**	**Non-converted**	**Odds ratio**	***p-value***
	**n = 36**	**n = 68**	**(95%CI)**	
	**(%)**	**(%)**		
DVT incidence	11 (30.6%)	5 (7.4%)	5.5 (1.7–17.6%)	0.028

**Figure 2:**
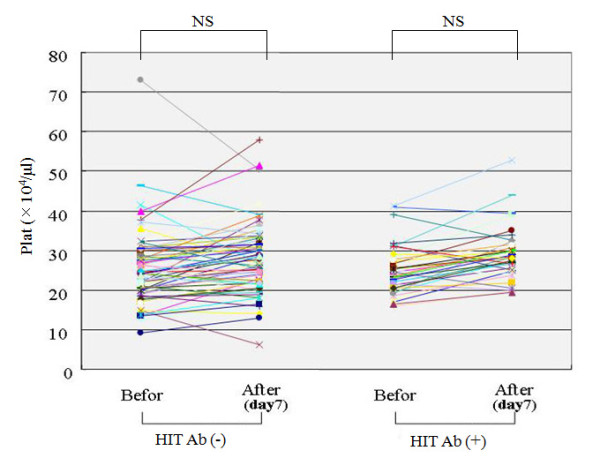
**Changes of platelet counts during the joint replacement treatment**. Platelet counts before operation and post-operation (post-operative day 7) in each patients with or without seroconvertion of HIT antibodies were shown.

**Figure 3:**
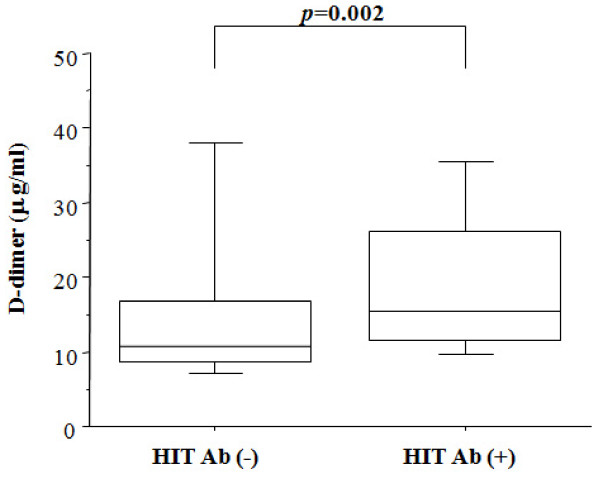
**D-dimer values in plasma of patients with or without seroconvertion of HIT antibodies at post-operative day 7**. Box plots indicate the mean, interquartile rage (25%–75%) and outliers (min, max).

There was no significant change in the platelet counts (before operation versus post-operative day 7) in the patients without HIT antibodies or in patients with HIT antibodies (Figure 2). One patient developed a low platelet count ( < 10 × 10^4^/ml). However, in this patient, no seroconversion of HIT antibody was observed. We also measured the coagulation marker, D-dimer, at postoperative day 7. The values of D-dimer differed when samples were separated into HIT antibody-positive and -negative groups (Figure 3). In the HIT antibody-positive group, the D-dimer values were significantly higher compared with those in the HIT antibody-negative group (HIT antibody (+); 21.5 ± 7.4 mg/ml versus HIT antibody (-); 17.5 ± 7.8 mg/ml). The preoperative clinical data were compared between patients with or without seroconversion for HIT antibody to identify the factors affecting seroconversion. There were no differences in the preoperative clinical data between the patients with or without HIT antibodies (Table 4).

**Table 4:** Patient characteristics and seroconversion of HIT Ab

**Characteristics**	**HIT Ab(-)** **n = 68**	**HIT Ab(+)** **n = 36**	***p-value***
Age (years), mean (SD)	68.5 ± 11.8	66.9 ± 12.3	0.50
Gender (male/female)	13/55	4/32	0.29
BMI (Kg/m^2^), mean (SD)	24.62 ± 4.31	25.71 ± 4.42	0.29
Metabolic diseases, n(%)	30(44.1%)	13(36.1%)	0.43
Hypertension			
Diabetes			
Hyperlipidemia			
History of thrombosis	-	1(2.8%)	-
TKA/THA	27/41	17/19	0.46
Primary disease	14/54	4/32	0.19
(RA/OA)			

HIT Ab, Anti-heparin platelet factor-4 antibody SD, standard deviation;

BMI, body mass index; TKA, total knee arthroplasty; THA, total hip arthroplasty;

RA, rheumatoid arthritis; OA, osteoarthritis.

## Discussion

Patients who have undergone orthopedic operations, such as total joint replacement of the lower extremity, are at high risk for thrombotic disease [1]. Without prophylaxis after TKA, the prevalence of DVT has been 41% to 85% [4]. In this study, we attempted to lower the incidence of DVT by combining a fixed dose of UFH with the intermittent pneumatic compression device. We found the incidence of symptomatic DVT after total joint replacement to be 15.4% overall (16/104) with distal (calf) thrombosis in all cases. The diagnosis of DVT is difficult in the absence of symptoms and consequently it is often under-diagnosed. Venography is the gold standard diagnostic method for DVT [12], but it is difficult to justify its use as a screening tool in all at-risk patients due to its invasive nature. The limitations of our study include the performance rate of contrast-enhanced computed tomography or venography, and only symptomatic DVT was surveyed. Therefore, the incidence of DVT in the present study cannot be compared to that of previous studies.

We could not confirm the association of DVT with underlying diseases, the location of the operation (knee or hip), or the presence of a thrombotic risk factor. However, we did find that the seroconversion of HIT antibody was significantly higher in post-operative patients with symptomatic DVT compared with those without symptomatic DVT. The values of D-dimer at post-operative day 7 were also significantly higher in patients with seroconversion for HIT antibody compared with those without seroconversion. These findings suggest that the presence of HIT antibodies could be linked to platelet activation, as demonstrated *in vitro*, [13] and that some patients having these antibodies develop thrombotic manifestations.

Type II HIT is a prothrombotic adverse drug reaction caused by platelet-activating antibodies that recognize PF4 bound to heparin (anti-PF4/heparin antibody) [14]. The PF4 becomes immunogenic when it is bound to heparin. Multi-molecular complexes of heparin, PF4, and IgG bind to the platelet Fc receptors resulting in platelet activation. Once these events are triggered, the prothrombotic risk remains for days to week, even after cessation of heparin therapy [13]. Therefore, type II HIT is an important adverse drug reaction induced by HIT antibodies that causes venous and arterial thrombosis [15]. Case reports and patient series have suggested that heparin-associated thrombosis might occur in patients with detectable HIT antibodies but in whom no thrombocytopenia is evident [16-19]. These findings suggest that a subset of HIT antibodies might exhibit a platelet activating profile without inducing thrombocytopenia. While confirmation of the presence of HIT antibodies is necessary, it is not sufficient for diagnosis of HIT since antibody formation occurs in a variety of clinical settings.

In HIT patients, the platelet counts begins to fall four or more days after the start of heparin therapy (typically between 5 and 10 days) [20]. Similarly, seroconversion of HIT antibodies occurs 5 to 10 days after the initiation of heparin therapy, except in patients with prior heparin therapy [21]. Consistent with these previous findings, our data demonstrated that seroconversion occurred in 34% of patients undergoing total joint replacement one week after the start of heparin. Our data suggest the clinical significance of HIT antibodies in DVT risk after joint replacement in the absence of thrombocytopenia.

A randomized clinical trial reported a significant difference in the occurrence of DVT between UFH and low molecular weight heparin (LMWH) in post-operative orthopedic patients [22-24]. More recently, a meta-analysis demonstrated that fondaparinux showed a major benefit by reducing VTE to a greater degree than LMWH in patients undergoing orthopedic surgery [25]. It was reported that HIT antibodies were generated at similar frequencies in patients treated with fondaparinux or LMWH [26]. However, *in vitro*, the antibodies induced by fondaparinux bind to PF4/LMWH and to PF4/UFHP, but not to PF4/fondaparinux. The few PF4/fondaparinux/Ab complexes are most likely inadequate to induce platelet activation via cross-linking [26]. The use of LMWH has not been approved or recommended for the prophylaxis of thromboembolism in Japanese orthopedic patients. Fondaparinux is now approved for major orthopedic surgery in Japan. Fondaparinux could be preferable to unfractionated heparin for DVT prophylaxis in patients undergoing total joint replacement. However, no evidence has yet been found to conclude that fondaparinux is effective in the prevention of VTE and is safe for Japanese orthopedics patients, and additional research is needed to determine the clinical benefit.

To summarize, in patients who undergo total hip or knee replacement with the use of UFH, the seroconversion of HIT antibodies contributes in part to the occurrence of symptomatic DVT after surgery. The measurement of HIT antibodies is warranted to estimate the risk of thromboembolism in orthopedic patients undergoing total joint replacement. Additional research is therefore needed to clarify the relationship between VTE risk and HIT antibodies in patients undergoing total joint replacement with the new prophylactic anti-coagulants.

## Conclusion

Seroconversion of HIT antibodies was associated with a risk of symptomatic DVT in patients undergoing total joint replacement under the prophylaxis of thromboembolism using heparin.

## Abbreviations

DVT: deep vein thrombosis; HIT: heparin-induced thrombocytopenia; LMWH: low molecular weight heparin; TKA: total knee arthroplasty; THA: total hip arthroplasty; PE: pulmonary embolism; UFH: unfractionated heparin; VTE: venous thromboembolism.

## Competing interests

The authors declare that they have no competing interests.

## Authors' contributions

TT, KM, YM carried out the immunoassays. KM, TH, GT, KT participated in the design of the study and performed the statistical analysis. SM, HS conceived the study, and participated in its design and coordination and helped to draft the manuscript. All authors read and approved the final manuscript.

## Pre-publication history

The pre-publication history for this paper can be accessed here:


